# Kikuchi Disease Masquerading as Lymphoma: Unravelling the Diagnostic Conundrum

**DOI:** 10.7759/cureus.110789

**Published:** 2026-06-13

**Authors:** Vidushi Saxena, Ram Shankar Mishra, Rohit Nayyar, Vivek Saxena

**Affiliations:** 1 Clinical Research, Max Institute of Medical Education, New Delhi, IND; 2 Internal Medicine, Max Super Speciality Hospital, Saket, New Delhi, IND; 3 Head and Neck Surgical Oncology, Max Super Speciality Hospital, Saket, New Delhi, IND; 4 Interventional Radiology, Max Super Speciality Hospital, Saket, New Delhi, IND

**Keywords:** fever of unknown origin, immunohistochemistry, kikuchi-fujimoto disease, lymphadenopathy, steroids

## Abstract

Kikuchi Disease is a rare, self-limiting autoinflammatory pathology, traditionally presenting with fever and cervical lymphadenopathy. Generalised lymphadenopathy remains an underreported presentation of this disease in the literature. We present the case of a 28-year-old woman who presented with fever of unknown origin along with unilateral cervical lymphadenopathy. PET-CT revealed generalised lymph node involvement with high tracer uptake values, creating the classic picture of lymphoma. Histopathology and immunohistochemistry analysis finally helped establish the diagnosis of Kikuchi disease. The non-specific presentation of this disease, coupled with its self-resolving nature, poses a significant diagnostic dilemma, underscoring the importance of a thorough workup and a high degree of clinical suspicion to guide accurate diagnosis.

## Introduction

Kikuchi-Fujimoto disease is a rare autoinflammatory disease of unclear etiology that leads to histiocytic necrotic lymphadenitis, primarily affecting young adults as well as the paediatric population [1]. This disease was first documented by Japanese pathologists Dr Kikuchi and Dr Fujimoto independently as two separate cases in 1972 [2,3]. Although its early descriptions are mainly in the Asian population, the disease has now been documented globally in various ethnic groups with debatable gender predominance [1]. With varying data from different regional studies, the exact global prevalence of this disease remains unclear.

Usually presenting with non-descript features such as low-grade fever and lymphadenopathy, this diagnosis presents a significant diagnostic challenge, especially in tuberculosis endemic regions [4]. Other noteworthy differentials for this disease include leprosy, infectious mononucleosis, autoimmune diseases, as well as T and B cell lymphomas [5]. This is a diagnosis of exclusion, with confirmation usually done by histopathological analysis. It usually portends a favourable prognosis with resolution of most cases within a few months of supportive management with a paltry recurrence rate of 3-4% [1]. The self-resolving nature of this disease, along with its vague presentation, often leads to it being underdiagnosed. 

Generalised lymphadenopathy is present only in a small cohort of patients (1-22%) [5]. Less than 10 cases with this rare manifestation have been reported in the literature, including cases from Kuwait [6], Germany [7], Korea [8], and the United States [9]. In India, fewer than five cases have been reported, including descriptions mainly in Western India by Nair et al. [10] and Kedar et al. [11]. No descriptions with this pattern of disease are present in the current literature in the North India region. This represents a noteworthy gap in the literature, which we hope to address with our case report.

Here, we discuss the case of a 28-year-old female patient from North India presenting with subacute fever and tender left cervical lymphadenopathy, later found to have generalised lymph node involvement on imaging. Diagnosed as Kikuchi disease on excisional biopsy, this case highlights a lesser-known phenotype of this disease, mimicking the clinicoradiologic picture of lymphoma.

## Case presentation

A 28-year-old married woman presented to the Internal Medicine outpatient department with the chief complaint of fever on and off for the past four weeks, with a maximum temperature of 100.3 ^o^F, according to the patient. She reported pain on the left side of her neck for one week, which was worse with neck movements. She also had throat pain for the last five days. The patient had previously consulted a local practitioner for her symptoms, during which she was managed conservatively. There was no significant past medical history, history of recent travel, or history of any other recent medical illness.

On examination, she was afebrile with stable vitals. A mildly tender swelling was palpated on the left side of the neck, measuring roughly 10 x 10 mm at the cervical level IIb with restricted neck movements. The rest of the systemic examination was unremarkable.

**Figure 1:**
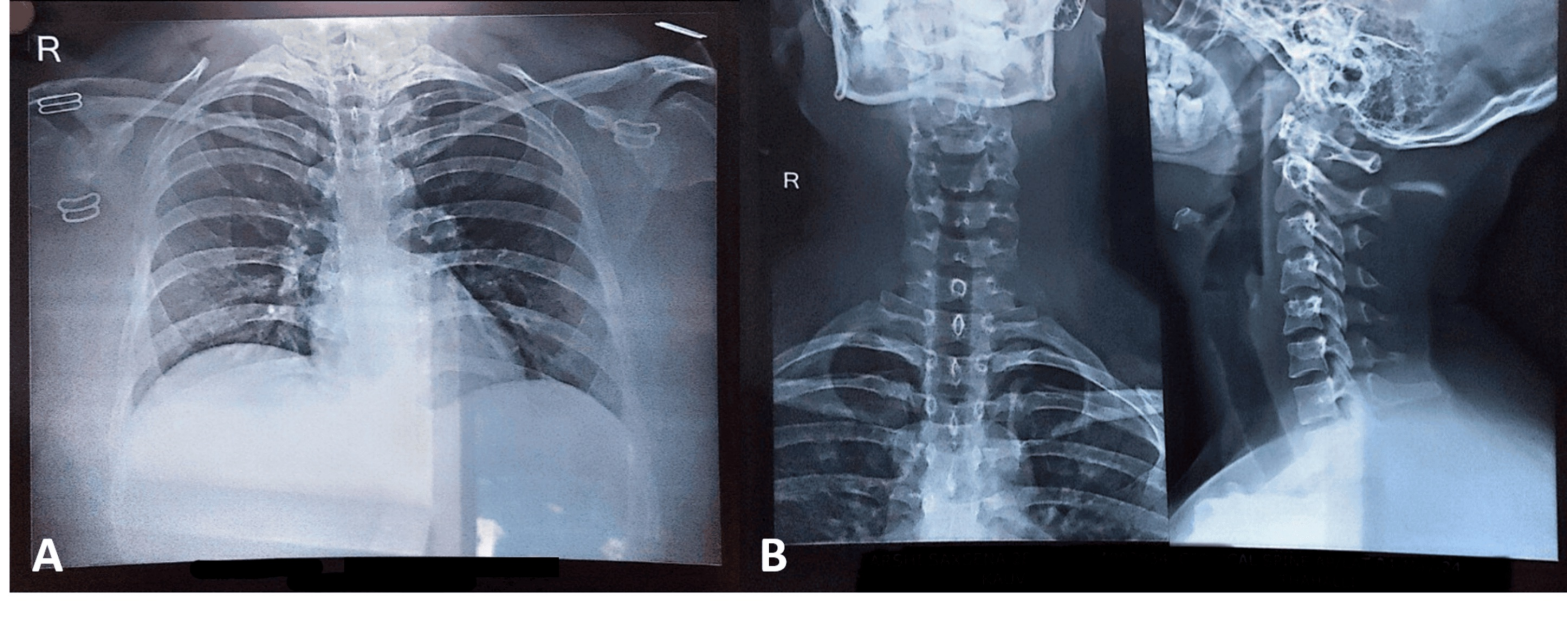
X-rays of (A) chest (posteroanterior view), (B) cervical spine (anteroposterior and lateral views) showing normal observations

Complete haemogram revealed normal hemoglobin (13.4 gm/dl) and platelet count (341 x 10^3^/uL). Total leukocyte count (TLC) was found to be 5.76 x 10^3^/uL with differential leukocyte count revealing monocytosis (12%) with raised inflammatory markers (C-reactive protein (CRP), 30.8 mg/dl, erythrocyte sedimentation rate (ESR), 74mm/hour). Liver and kidney function tests were within normal limits. Blood cultures, QuantiFERON-TB Gold test (QIAGEN N.V., Venlo, Netherlands), malaria testing with thick and thin smears, dengue NS1 (non-structural protein 1) with IgG and IgM, as well as Typhidot IgM and IgG, were negative. Viral markers for HIV, hepatitis B surface antigen, hepatitis C, Epstein-Barr virus (EBV), and cytomegalovirus (CMV) were also found to be negative. X-rays of the chest and cervical spine were normal (Figure 1).

**Figure 2:**
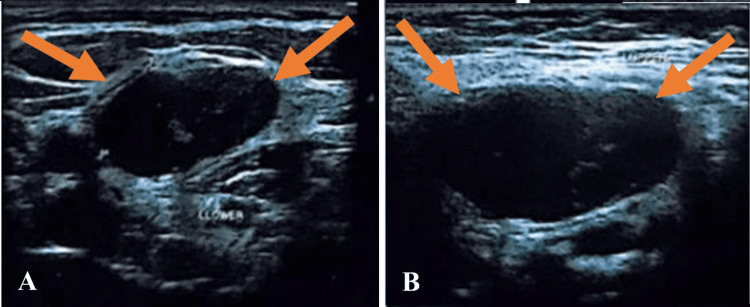
Enlarged left cervical lymph nodes with preserved fatty hilum with largest measuring 2.3 x 1.3 cm at level Ib (A) Left lower cervical lymphadenopathy; (B) Left upper cervical lymphadenopathy

An ultrasound of the neck region was done to evaluate the left-sided neck swelling. Findings revealed multiple enlarged lymph nodes on the left side at levels Ib, II, III, IV, and V, with the largest measuring 2.3 x 1.3 cm at level Ib with morphological features suggestive of infective etiology (Figure 2). 

Repeat blood testing showed blood cell counts to be within normal limits (hemoglobin, 12gm/dl, platelet count, 290 x 10^3^/uL, TLC, 4.15 x10^3^/uL) with uptrending inflammatory markers (CRP, 38 mg/dl, ESR, 80 mm/hour). Repeat blood cultures were negative. Fine needle aspiration cytology (FNAC) from the left cervical lymph node was performed, which revealed richly cellular smears with scattered lymphoid cells, immunoblasts, and histiocytes with no evidence of epithelial granulomas or necrosis, suggestive of reactive lymphoid hyperplasia.

Autoimmune panel including antinuclear antibody (ANA), antineutrophil cytoplasmic antibodies (ANCA), and rheumatoid factor (RF) along with IgM and IgG for rickettsia, scrub typhus, and bartonella were sent, which came back negative. Repeat hemogram was significant for progressive normocytic anemia with hemoglobin levels downtrending to 11.2 gm/dl with consistent upward trend of inflammatory markers (ESR, 84 mm/hour, CRP, 42 mg/l) (Table 1). 

**Table 1:** : Longitudinal lab value trends with infectious and autoimmune workup findings MCV: mean corpuscular volume; WBC: white blood cell; ESR: erythrocyte sedimentation rate; CRP: C-reactive protein; ALT: alanine aminotransaminase; AST: aspartate aminotransferase; ALP: alkaline phosphatase; TB: tuberculosis; HBsAg: hepatitis B surface antigen; PF/PV: *Plasmodium falciparum*/*Plasmodium vivax*; ANA: antinuclear antibody; ANCA: antineutrophil cytoplasmic antibodies; RF: rheumatoid factor; ELISA: enzyme-linked immunosorbent assay; IFA: indirect immunofluorescent assay

Variable	Day 1	Day 5	Day 9	Reference Range
Haemoglobin (gm/dL)	13.4	12	11.2	12-16 gm/dL
MCV (fL)	83.88	82.1	81.5	78-96 fL
Platelet count (10^3^/uL)	341	290	328	150-450 x 10^3^/uL
Total WBC count (10^3^/uL)	5.76	4.15	6.19	4-11 x 10^3^/uL
Neutrophils (%)	54.8	53	55	50-75%
Lymphocytes (%)	30.4	36	33	20-40%
Monocytes (%)	12	10	10	02-10%
Eosinophils (%)	2.5	01	02	01-06%
Basophils (%)	0.1	00	00	00-01%
ESR (mm/hr)	74	80	84	02-18 mm/hr
CRP (mg/L)	30.8	38	42	0-6 mg/L
ALT (IU/L)	39	43	-	7-55 IU/L
AST (IU/L)	31	34	-	8-48 IU/L
ALP (IU/L)	98	102	-	40-129 IU/L
Serum Creatinine (mg/dL)	0.6	0.7	-	0.6-1.1 mg/dL
Blood Urea Nitrogen (mg/dL)	10	12	-	7-20 mg/dL
Blood Culture	Sterile	Sterile	Sterile	-
Quantiferon TB Gold test (ELISA)	Negative	-	-	Negative:<0.35 IU/mL
Blood smear for malarial parasite	Not detected	-	-	-
Malaria Antigen PF/PV	Negative	-	-	Negative
Dengue IgM/IgG	Negative	-	-	Negative
Dengue NS1 Antigen	Negative	-	-	Negative: <0.8
Typhidot IgM/IgG	Negative	-	-	Negative
HIV 1 & 2 Antibody test	Non reactive	-	-	Non reactive: <1.00 index
HBsAg serology	Non reactive	-	-	Non reactive: <1.00 index
HCV Antibody serology	Non reactive	-	-	Non reactive: <1.00 index
EBV IgM serology	Negative	-	-	Negative: <0.90 index
CMV IgM serology	Negative	-	-	Negative: <0.90 index
Rickettsia IgM (ELISA)	-	-	Negative	Negative: < 0.9 index
Scrub typhus IgM (ELISA)	-	-	Negative	Negative: <0.9 index
Bartonella IgM (IFA)	-	-	Negative	Negative: <1:20 titre
ANA			Negative	Negative: <1:80 titre
ANCA (pANCA/cANCA)			Negative	Negative: <1:20 titre
RF			Within normal limits	<20 IU/ml

A whole-body PET-CT was done to evaluate this fever of unknown origin. The findings revealed widespread fluorodeoxyglucose (FDG) avid lymphadenopathy, with many of them showing a high standardized uptake value (SUV). In the head and neck region, multiple FDG-avid lymph nodes were seen in the left supraclavicular region and bilateral cervical region with SUVmax reaching up to 6.40. In the thorax, an FDG-avid lymph node of 19 x 12 mm (SUVmax 3.25) was seen in the right anterior cardiophrenic fat pad region. The abdomen and pelvis showed an FDG-avid conglomerated enhancing and necrotic lymph nodal mass at the porta-precaval region with aggregate size measuring about 39 x 30 mm (SUVmax 8.83). FDG-avid lymph nodes were also seen adjacent to the gallbladder neck and celiac trunk origin, along with multiple FDG-avid lymph nodes in the retroperitoneum, including pre-paraaortic, aortocaval region, retrocaval region, and left paraaortic region, with the largest aortocaval node measuring 15 × 12 mm (SUVmax 6.93). Additionally, focal areas of FDG avidity were also seen in multiple dorsolumbar vertebral bodies (maximal at L5 with SUVmax 3.14) and in the sacral body (SUVmax 3.31). Fatty liver changes with mild hepatomegaly and mild splenomegaly were noted as well.

Based on the aforementioned findings, the diagnosis of lymphoma was suspected. A diagnostic left level II and V cervical lymph node excisional biopsy was done for diagnostic confirmation.

On histopathology, sections from all lymph nodes revealed preserved architecture with retained follicles and sinuses. The lymphoid infiltrate comprised small mature lymphocytes, plasma cells, and immunoblasts. Lymph nodes demonstrated nodular and sheet-like infiltration by histiocytes and focal fibrosis along with a small necrotising granuloma. No giant cells were seen, and Ziehl-Neelsen (ZN) staining was negative for acid-fast bacilli.

**Figure 3:**
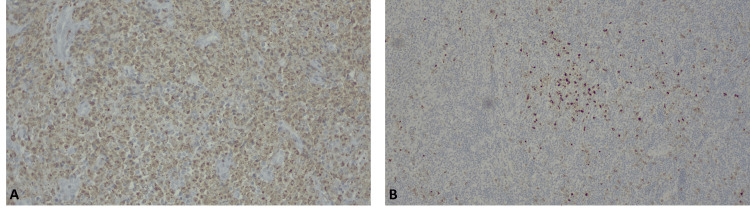
Lymphoid infiltrate consisting of CD68 positive histiocytes (A) along with occasional characteristic MPO positive cells (B)

**Figure 4:**
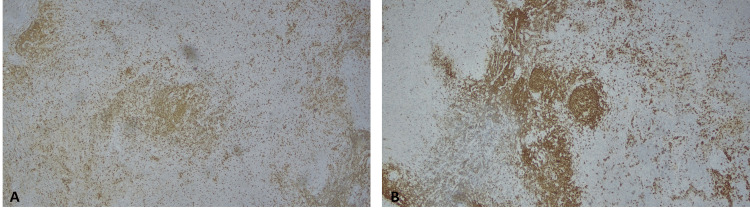
Normal lymph node architecture with T cells and B cells present in appropriate areas of lymph node (A) CD3 highlighting reactive T cells present in appropriate areas of lymph node; (B) CD20 highlighting germinal centres and reactive B cells seen in appropriate areas of lymph node

**Figure 5:**
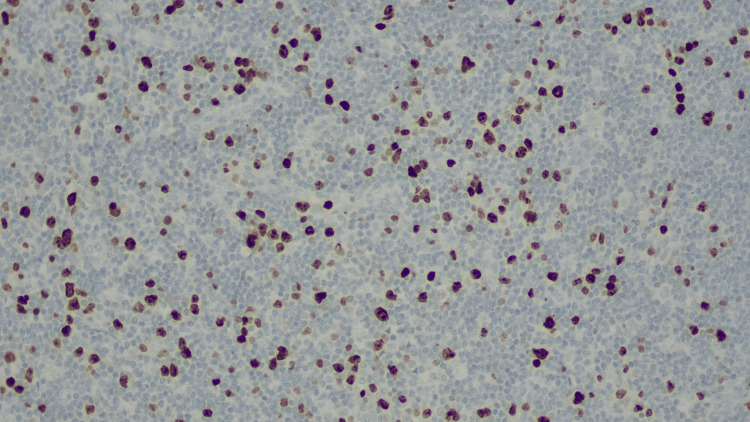
Ki67 proliferation index 70% at histiocytic proliferation sites compared to 25-30% in other sites

Immunohistochemistry analysis showed histiocytic cells to be weakly positive for CD45, strongly positive for S100 and CD68, with some cells positive for MPO (Figure 3). The cells were negative for CD 30, CD 3, CD 20, CD 19, and Alk-1. CD 3 and CD 20 marked appropriate areas of the lymph node (Figure 4). Immunoblasts were positive for CD30, CD21, and CD23, highlighting the follicular dendritic cells. Ki-67 labelling index was 70% at the histiocytic proliferation site compared to 25-30% in other sites (Figure 5).

Preserved architecture of the lymph node with MPO and CD68 positive histiocytic proliferation with strong S-100 positive indicative of their plasmacytic dendritic character confirmed the diagnosis of Kikuchi disease in the proliferative phase [12].

The patient was managed using nonsteroidal anti-inflammatory drugs (NSAIDs). Additionally, in lieu of generalised lymphadenopathy, a short tapering course of prednisolone was also given. The patient reported resolution of lymphadenopathy and no persisting symptoms after three months. 

## Discussion

Kikuchi disease was first elucidated in Japan as two separate cases describing follicular reticular cell hyperplasia and necrotizing lymphadenitis, respectively [2,3]. Although this disease has been well documented in the literature, the exact mechanism of this disease is still unclear. Human leukocyte antigen (HLA) type II linkage, especially with subtypes HLA-DPB1 and HLA-DPA1, has been found, making an autoimmune mechanism likely [13]. Some studies have attributed this disease to a viral infection that triggers self-limiting inflammation. This is postulated to be caused by an aberrant type I interferon response as evidenced by CD8+ T cells and CD123+ plasmacytoid dendritic cell predominant inflammation seen in this disease [14].

This disease has not only been linked to viral infections like EBV, CMV, HIV, and paramyxoviruses, but also to bacterial infections such as *Yersinia*, *Streptococcus pneumoniae*, and *Bartonella* [15]. Associations have also been seen with autoimmune diseases such as systemic lupus erythematosus (SLE) and Sjögren’s disease, as well as with vaccinations including HPV, influenza, Japanese encephalitis as well as COVID 19 vaccination [15,16]. 

Kikuchi disease classically presents with fever and cervical lymphadenopathy. Other common symptoms of this disease include fatigue, erythematous rash, and arthralgias [15]. The central nervous system can also be involved in a minority of patients, mostly affecting the brain as aseptic meningitis and encephalitis or presenting with neuroophthalmologic complications like optic neuritis [17]. Our case presented with low-grade fever and tender left cervical lymphadenopathy, in line with the textbook presentation of Kikuchi disease. This vague constellation of symptoms presented a significant diagnostic challenge, particularly in a TB endemic region like ours, with several contending differentials including infectious and autoimmune pathologies.

Detailed diagnostic workup did not yield any significant findings. Laboratory trends revealed initial monocytosis, progressive normocytic anemia, and uptrending inflammatory markers, despite persistent negative cultures, serologies, and autoimmune markers. These findings are consistent with lab variations commonly documented in Kikuchi disease. An analysis of 244 cases of Kikuchi disease by Kucukardali et al. demonstrated raised ESR in 40% of cases, anemia in 23% of cases, and leucopenia in 43% of cases [18].

In our case, further evaluation with whole-body PET- CT revealed widespread generalised lymphadenopathy with multiple FDG-avid lymph nodes with high SUVmax values in the cervical region, mediastinum, as well as retroperitoneum. Presentation with generalised lymphadenopathy, as in our case, has been reported in only a small cohort of patients (1-22%), representing a lesser-known phenotype of this rare condition [5]. Limited documentation of this presentation in the literature might be attributed to under-imaging of patients. A large single-centre study in Taiwan, which analysed over 60 patients with Kikuchi disease using CT scans, demonstrated extracervical lymphadenopathy in 30-40% of cases, with most cases involving the abdomen and minimal involvement of the mediastinum [19].

Widespread lymphadenopathy with high uptake values and extranodal involvement of vertebral bodies, along with the clinical findings of persistent constitutional symptoms and cervical lymphadenopathy, pointed towards the diagnosis of malignant lymphoma. Interestingly, Kikuchi disease has also been linked to acute lymphomas, presumably due to the HLA class II linkages in both diseases, making histologic and immunohistochemistry analysis imperative in such cases to rule out concurrent lymphoma [20].

Histopathology and immunohistochemistry evaluation of our case revealed preserved lymphoid architecture with infiltration of CD68-positive histiocytes. Histiocytes with strong S-100 positivity, indicating plasmacytic dendritic character and characteristic MPO-positive phenotype, were deemed indicative of Kikuchi disease specifically in the proliferative phase [12]. Negative hallmark markers with no evidence of atypical cells or disruption of lymph node architecture, along with a high Ki-67 proliferation index only seen in areas of histiocytic proliferation, argued against the diagnosis of lymphoma in our case. 

Despite inconclusive findings of reactive hyperplasia on FNAC, the diagnosis of Kikuchi disease in our case serves as a powerful reminder of the utility of histopathology and immunohistochemistry analysis in definitively establishing the diagnosis, reiterating the primarily adjunctive role of FNAC for this diagnosis.

As of now, there are no treatment guidelines for this disease. Management is usually done using NSAIDs for symptomatic management with the addition of steroids or immunomodulators in severe generalised disease with extranodal involvement [15]. Symptom resolution on addition of a short tapering course of steroids in our case reinforces that generalized lymphadenopathy is predictive for a more severe form of disease, often requiring escalation of treatment beyond mere supportive management [19]. 

## Conclusions

The obscure presentation of Kikuchi disease can mimic the clinico-radiologic presentation of lymphoma. A high degree of clinical suspicion and a thorough workup are required to diagnose this condition. Histopathology and immunohistochemistry analysis are imperative for diagnosis, with FNAC findings only considered supportive.
